# Exploring inter-ethnic and inter-patient variability and optimal dosing of osimertinib: a physiologically based pharmacokinetic modeling approach

**DOI:** 10.3389/fphar.2024.1363259

**Published:** 2024-03-04

**Authors:** Feng Liang, Yimei Zhang, Qian Xue, Na Yao

**Affiliations:** Bethune International Peace Hospital, Shijiazhuang, China

**Keywords:** osimertinib, PBPK model, ethnic differences, inter-patients variability, optimal dosing regimens

## Abstract

**Purpose:** This study aimed to develop and validate a physiologically based pharmacokinetic (PBPK) model for osimertinib (OSI) to predict plasma trough concentration (C_trough_) and pulmonary EGFRm^+^ (T790M and L858R mutants) inhibition in Caucasian, Japanese, and Chinese populations. The PBPK model was also utilized to investigate inter-ethnic and inter-patient differences in OSI pharmacokinetics (PK) and determine optimal dosing regimens.

**Methods:** Population PBPK models of OSI for healthy and disease populations were developed using physicochemical and biochemical properties of OSI and physiological parameters of different groups. And then the PBPK models were validated using the multiple clinical PK and drug-drug interaction (DDI) study data.

**Results:** The model demonstrated good consistency with the observed data, with most of prediction-to-observation ratios of 0.8–1.25 for AUC, C_max_, and C_trough_. The PBPK model revealed that plasma exposure of OSI was approximately 2-fold higher in patients compared to healthy individuals, and higher exposure observed in Caucasians compared to other ethnic groups. This was primarily attributed to a lower CL/F of OSI in patients and Caucasian. The PBPK model displayed that key factors influencing PK and EGFRm^+^ inhibition differences included genetic polymorphism of CYP3A4, CYP1A2 expression, plasma free concentration (f_up_), albumin level, and auto-inhibition/induction on CYP3A4. Inter-patient PK variability was most influenced by CYP3A4 variants, f_up_, and albumin level. The PBPK simulations indicated that the optimal dosing regimen for patients across the three populations of European, Japanese, and Chinese ancestry was OSI 80 mg once daily (OD) to achieve the desired range of plasma C_trough_ (328–677 nmol/L), as well as 80 mg and 160 mg OD for desirable pulmonary EGFRm^+^ inhibition (>80%).

**Conclusion**: In conclusion, this study’s PBPK simulations highlighted potential ethnic and inter-patient variability in OSI PK and EGFRm^+^ inhibition between Caucasian, Japanese, and Chinese populations, while also providing insights into optimal dosing regimens of OSI.

## 1 Introduction

Lung cancer is the leading cause of cancer-related deaths globally, contributing to approximately 18.4% of total cancer mortality (Thandra et al., 2021). The majority of lung cancer cases (over 80%) are classified as non-small cell lung cancer (NSCLC) (Fujimoto et al., 2019). Among NSCLC patients, the Epidermal Growth Factor Receptor (EGFR) has been identified as an effective therapeutic target (Liu et al., 2017). EGFR mutations (EGFRm^+^) are crucial oncogenic driver alterations in NSCLC, occurring in approximately 10%–15% of cases in Caucasians and with a higher frequency of up to 50% among East Asians. (Low et al., 2023). Exon 19 deletions and L858R mutations are the most common types, accounting for 90% of all EGFRm^+^ (Low et al., 2023). These EGFRm^+^ mutations still are highly responsive to EGFR tyrosine kinase inhibitors (TKIs) (Low et al., 2023). In 2004, the T790M mutation in exon 20 of EGFR was first discovered and described (Yasuda et al., 2013; Low et al., 2023). Although T790M mutations only account for approximately 5% of all EGFRm^+^ cases, around 50% of these mutations develop resistance to TKIs (Food and Drug Administration FDA, 2015).

Osimertinib (OSI) is an oral, third-generation EGFR TKI developed by AstraZeneca specifically for the treatment of NSCLC (Greig, 2016). It targets the EGFR T790M mutation present in NSCLC patients who have developed resistance to other EGFR TKIs. The approved dosage strength of OSI tablets for patients is 80 mg (Food and Drug Administration, 2022). The primary metabolic pathway of OSI is through CYP3A, with minor contributions from CYP1A2 and CYP2C9 (Food and Drug Administration FDA, 2015). Furthermore, OSI acts as a competitive inhibitor of CYP3A4 and significantly induces the expression of CYP3A4 (Food and Drug Administration FDA, 2015).

Variability in the plasma area under the curve (AUC) of AZ5104 (metabolite of OSI) has been observed among different ethnic groups, with a 10%–23% decrease in Asian NSCLC patients compared to Caucasian patients (Brown et al., 2017). Moreover, there is a high degree of variability in OSI pharmacokinetics (PK) of inter-patients, with a percentage coefficient of variation (%CV) exceeding 50% and, in some cases, even surpassing 80% (Planchard et al., 2016). The relationship between exposure and response demonstrates that optimal efficacy and safety are achieved when the plasma trough concentration (C_trough_) of OSI falls within the range of 164–338 ng/mL (equivalent to 328–677 nM) (Abu Hamdh and Nazzal, 2023), resulting in longer progression-free survival (PFS) and potentially mitigating certain adverse events. However, it is suggested that the pulmonary EGFRm^+^ inhibition could serve as a more valuable indicator for clinical efficacy, as it reflects the two EGFR m^+^ engagement in the targeted tissue.

The variability in the PK of OSI can be influenced by genetic polymorphism of the CYP3A4 enzyme, which plays a key role in OSI metabolism. Differences of CYP3A4 metabolism in ethnic groups can also contribute to PK variability. Another significant enzyme involved in OSI metabolism is CYP1A2 (Food and Drug Administration FDA, 2015). It has been demonstrated that smoking can induce CYP1A2 expression, leading to 1.55-fold higher enzyme activity in smokers compared to non-smokers (Dobrinas et al., 2011). Consequently, PK variability of OSI may occur in both smokers and non-smokers. Two clinical studies have indicated a statistically significant correlation between smoking and clinical efficacy (Rodier et al., 2022; Abu Hamdh and Nazzal, 2023). Additionally, auto-inhibition and induction effects of OSI on CYP3A4 can cause differences in PK between multiple doses (MD) and single dose (SD). OSI has a high plasma albumin binding rate of approximately 99%, with a 10-fold difference in the remaining fraction (f_up_) at different concentrations (Food and Drug Administration FDA, 2015). Variations in f_up_ can result in greater PK variability. Moreover, several clinical studies have demonstrated that the level of plasma albumin, which predominantly binds to OSI (Food and Drug Administration FDA, 2015), can influence the distribution and clearance of OSI in NSCLC patients, and have a statistically significant correlation with the efficacy of OSI in NSCLC patients (Yokota et al., 2022; Ishikawa et al., 2023).

The current literature includes seven the clinical PK studies on OSI, but a systematic assessment of variability in systemic exposure has not been conducted. To this end, a physiologically based pharmacokinetic (PBPK) model was developed and validated in three populations (Caucasian, Japanese, and Chinese). The PBPK model is then used to evaluate inter-ethnic and inter-patients differences in plasma PK and the inhibition of pulmonary EGFRm^+^ (T790M and L858R). Furthermore, this study utilized the PBPK model to analyze the impact of several major sensitive factors, including CYP3A4 genetic polymorphism, CYP1A2 activity, auto-inhibition and induction, f_up_, and albumin level, on the plasma C_trough_ of OSI and the inhibition of EGFRm_+_. Finally, the PBPK model was employed to determine an optimal dosing regimen for OSI in different populations based on the plasma C_trough_ and pulmonary EGFRm^+^ inhibition threshold value for efficacy and safety.

## 2 Methods

### 2.1 Development and validation of the PBPK model in three ethnic populations

The PBPK models for healthy and diseased population in three ethnic groups were developed using the PK-Sim^®^ (Version 10.0, Bayer Technology Services, Leverkusen, Germany).

#### 2.1.1 The PBPK model for healthy population

Table 1 provides a summary of the modeling parameters utilized in the PBPK model (Dixon et al., 2003; Barter et al., 2013; Dickinson et al., 2016; Pilla Reddy et al., 2018; Schwenger et al., 2018; Alsmadi et al., 2021; An et al., 2021; Hashino et al., 2023; Pharmaceuticals and Medical Devices Agency PMDA, 2023). Rodgers and Rowland, and PK-Sim’s standard methods provided the human tissue distribution and cellular permeability estimate for OSI. While Rodgers and Rowland’s method can estimate the human tissue-to-plasma partition coefficient, it is preferable to incorporate experimental values into the PBPK model, particularly for the target tissue. The lung-to-plasma partition coefficient (K_lu,p_) has been experimentally determine for the distribution of OSI in lung tissue. Based on average tissue-to-blood ratios at 1 h and 6 h from reference (Dickinson et al., 2016), the experimental K_lu,*p*_-value was manually entered into this model.

**TABLE 1:** OSI-specific and physiological parameters used in the development of PBPK model.

Parameters (Units)		Values used in the model		Source and comments
		Healthy	Diseased	
MW(g·mol^-1^)		499.6		Chemspider
pKa (Base)		9.5, 4.4		Pilla Reddy et al. (2018)
Log P		5.45		
Solubility (mg·mL^-1^)		3.1(Water)		Pharmaceuticals and Medical Devices Agency PMDA (2023)
P_eff_ (✕10^–4^ cm⋅s^-1^)		0.187		Pilla Reddy et al. (2018)
f_up_		0.013	0.019	0.013 from the Ref. (Pilla Reddy et al., 2018) and 0.019 was calculated using Eq. 1
Rbp		1.0	1.1	1.0 from the Ref. (Pilla Reddy et al., 2018) and 1.1 was calculated using equation(2)/(3)
CYP1A2 CL_int,u_ (μL/min/pmol)		0.52		Pilla Reddy et al. (2018)
CYP2A6 CL_int,u_ (μL/min/pmol)		0.37		
CYP2C9 CL_int,u_ (μL/min/pmol)		0.48		
CYP2E1 CL_int,u_ (μL/min/pmol)		0.11		
CYP3A4 CL_int,u_ (μL/min/pmol)		0.73		
CYP3A5 CL_int,u_ (μL/min/pmol)		0.21		
CL_R_(L/h)		GFR*f_up_		Default calculation in PK-Sim
Liver volume (L)		Caucasian: 2.38, Japanese: 1.91, Chinese: 2.16		Default value of ethnic population in PK-Sim
GET (min)		15	120	Default 15 min; 120 min was optimized from 190 min (Ref. (Alsmadi et al., 2021))
K_lu,p_		28.5		Mean value form the Ref. (Dickinson et al., 2016)
K_p_ scale		1.5		Optimized based on better tissue distribution description
Hematocrit		0.47	0.33	Default value of 0.47. 0.33 from the Ref. (Dixon et al., 2003)
Albumin (g/dl)		0.45	0.31(Caucasian,20%CV; Japanese and Chinese, 30%CV)	Ref. (Dixon et al., 2003; Hashino et al., 2023)
Concentration (μM/L liver tissue)	CYP1A2	1.80	1.33	Default value for healthy population. Obtained for CYP2C9 and CYP3A4 for diseased population from Ref. (Alsmadi et al., 2021); CYP1A2/2A6/2E1/3A5 concentrations were calculated with 20%–33% reductions based on Ref (Schwenger et al., 2018)
	CYP2A6	2.72	1.90	
	CYP2C9	3.84	3.20	
	CYP2E1	1.96	1.37	
	CYP3A4	4.32	3.02	
	CYP3A5	0.04	0.028	
Abundance (pmol/mg protein)	CYP1A2	Caucasian: 52, Japanese: 31.8, Chinese: 42		Obtained from Ref (Barter et al., 2013; An et al., 2021)
	CYP2A6	Caucasian: 36, Japanese:11.5, Chinese:14		
	CYP2C9	Caucasian: 73, Japanese: 59.2, Chinese: 60		
	CYP2E1	Caucasian: 61, Japanese: 36, Chinese: 70.5		
	CYP3A4	Caucasian: 137, Japanese:112, Chinese:120		
	CYP3A5	Caucasian: 116, Japanese:27.8, Chinese:99		
K_i_ CYP3A4/5 (μM)		2.55		Pilla Reddy et al. (2018)
EC_50_ CYP3A4 (μM)		0.12		
E_max_ CYP3A4		10.8		

MW, molecular weight; pKa (Base), Base dissociation constant; Log P, lipophilicity; f_up_, Free fraction in plasma; R_bp_, Blood-to-plasma concentration ratio; CL_int,u_, Intrinsic clearance; CL_R_, renal clearance; GET, gastric emptying time; GFR; K_lu,p_, lung-to-plasma partition coefficient; K_p_ scale:Organ-to-plasma partition coefficient; K_i_, 50% maximal inactivation rate; EC_50_, Inducer concentration required to achieve 50% inductive effect; E_max_, Maximum inductive effect for CYP3A4.

Additionally, the K_p_ scale was optimized to 1.5 to better represent the tissue distribution of OSI. The involvement of kidney transporters or tubules in the uptake or excretion of OLA was not reported. Thus, the fraction of glomerular filtration rate (GFR) was set as 1.0. The renal clearance (CL_R_) was estimated by the glomerular filtration rate (GFR)✕f_up_ method using PK-Sim. Hepatic clearance of OSI was estimated by considering six metabolism enzymes.

When considering inter-ethnic differences, the enzyme abundances and liver volume are the most commonly considered factors. In this study, default liver volumes of 2.38 L for Caucasians, 2.16 L for Japanese, and 1.91 L for Chinese populations were utilized. Additionally, Table 1 presents the abundance of six metabolizing enzymes in different ethnic groups based on literature data from references (Barter et al., 2013; An et al., 2021). These enzyme abundances are taken into account to capture potential inter-ethnic variations in OSI metabolism.

#### 2.1.2 The PBPK model for diseased population

In the diseased PBPK model, the same model structures as the healthy PBPK model were used, with some modifications specific to NSCLC populations based on previous published papers. Firstly, the gastric emptying time (GET) parameter was optimized to 120 min, different from the previously used value of 190 min, according to reference (Alsmadi et al., 2021). This adjustment was made based on the peak time that better aligned with the clinical PK data. The concentrations of CYP3A4 and CYP2C9 enzymes were assigned based on reference (Alsmadi et al., 2021). The concentration of CYP1A2 in cancer patients was calculated to be 1.33 μM, taking into account a 26% downregulation relative to the healthy population as reported in reference (Schwenger et al., 2018). For the remaining three metabolizing enzymes, their expression levels in patients were calculated assuming a ratio of 0.7 compared to the healthy population. The plasma albumin levels were set at 0.45 g/dL in the healthy population and 0.31 g/dL in the diseased population, based on information from published papers (Dixon et al., 2003; Hashino et al., 2023). Finally, the f_up_ and blood-to-plasma concentration ratio (R_bp_) in NSCLC patients were determined using Eqs 1–3:

$$f_{\text{up}}\left(\text{patients}\right)=1/(1+((1-f_{\text{up}}(\text{healthy}))\times \left[P\right]\left(\text{patients}\right))/(\left[P\right]\left(\text{healthy}\right)\times f_{\text{up}}\left(\text{healthy}))\right)$$ (1)

Where [P](patients) and [P](healthy) represent the plasma albumin levels in patients and healthy population, respectively.

$$R_{\text{bp}}\left(\text{patients}\right)=1+\text{Hct }\times \left(f_{\text{up}}\left(\text{healthy}\right)*K_{\text{puBC}}-1\right)$$ (2)

Where R_bp_ (Patients) represents the patients’ blood-to-plasma concentration ratio in; Hct represents the hematocrit value; K_puBC_ is affinity of blood cells to the OSI. K_puBC_ is estimated by:

$$K_{\text{puBC}}=(\text{Hct}-1+\text{Rbp}\left(\text{healthy}\right))/\left(\text{Hct}\times f_{\text{up}}\left(\text{healthy}\right)\right)$$ (3)

In the diseased PBPK model, the remaining modeling parameters were assumed to be the same as those used for the healthy population.

#### 2.1.3 Validation of the PBPK model

To validate the predictive performance of the PBPK model, multiple clinically observed PK data of OSI in Caucasian (Planchard et al., 2016; Vishwanathan et al., 2018a; Harvey et al., 2018; Grande et al., 2019; Vishwanathan et al., 2019), Japanese (Planchard et al., 2016; Fujiwara et al., 2023), and Chinese (Zhao et al., 2018) populations were utilized. The model’s accuracy was assessed by comparing the coincidences between predicted and observed PK variables such as area under the curve (AUC) and maximum concentration (C_max_) in healthy and diseased populations after a single dose and repeated doses.

In general, an accurate prediction model is considered acceptable when the ratios of these PK variables between prediction and observation fall within the range of 0.5–2.0. This criterion ensures that the model’s predictions are reasonably close to the observed values, indicating a good level of accuracy and reliability in the PBPK model’s performance for OSI across different ethnic populations.

The fraction metabolic contribution for CYP3A4 on OSI is estimated to be 47.6%, and OSI can inhibit and reduce activity of CYP3A4. Hence, to ensure the accuracy of CYP3A4 metabolism contribution in the PBPK model, the PK interactions between OSI and itraconazole (ITR) as well as rifampicin (RIF) were simulated in Asian populations. The PBPK model and interaction parameters for ITR and RIF were obtained from published papers (Gao et al., 2023). Based on the clinical drug-drug interaction (DDI) study (Vishwanathan et al., 2018b), The DDI simulation between OSI and ITR involved administering OSI at a dose of 80 mg once daily (OD) on day 1 and 10, while ITR was administered at a dose of 200 mg twice daily from day 7 to day 19. Similarly, in the DDI simulation between OSI and RIF, OSI was dosed at 80 mg OD on day 1 and 29, while RIF was administered at a dose of 600 mg OD from day 7 to day 29. The simulated fold difference for the DDIs of OSI with ITR and RIF were then compared with clinically observed ratios. By evaluating the agreement between the simulated and observed data for these DDIs, the reliability and predictive performance of the CYP3A4 metabolism parameters can be verified.

### 2.2 Sensitivity analysis

The modeling parameters that were optimized and identified as having potentially significant effects on PK variables of OSI (under the steady-state concentration-time curve: AUC_ss_, steady-state peak concentration: C_ss,max_ and plasma C_trough_) were chosen for the sensitivity analysis. These specific parameters include: 1) f_up_, 2) Rbp, 3) GET, 4) Albumin level, 5) CYP CL_int,u_, 6) CYP3A4 auto inhibition and induction parameters, 7) CYP enzyme expression, and 8) liver volume.

To evaluate the influences of the selected parameters on PK variables, each parameter’s value was altered by ±20% during the sensitivity analysis. The sensitivity coefficient (SC) was then calculated using the following Eq. 4 (Li et al., 2020):

SC=∆Y/Y÷∆P/P (4)

Where ∆Y is the alteration of predicted PK variables; Y is the initial value of predicted PK variables; ∆P is the alteration of model parameters; P is initial value of assessed parameters. If the absolute value of SC is above 1.0, it indicates that this parameter has a significant influence on the predicted PK variables.

### 2.3 Plasma C_trough_ and EGFRm^+^ inhibition prediction

The developed PBPK model was utilized to simulate the plasma C_trough_ and EGFRm^+^ inhibition time profiles for three ethnic populations with NSCLC. The virtual population’s demographic characteristics and dosage regimes were based on relevant clinical papers (Table 2). Each simulation consisted of ten virtual subjects. The estimation of EGFRm^+^ (T790M and L858R) inhibition in plasma or lung time profiles was achieved through the utilization of the following Eqs 5–7:

$$\frac{dOEm}{dt}=k_{on}{\times C}_{lung}\times {Em}_{free}-k_{off}\times OEm$$ (5)

$$\frac{d{Em}_{free}}{dt}={\left({Em}_{0}-{Em}_{free}\right)\times k_{turnover}-k}_{on}{\times C}_{lung}{\times Em}_{free}+k_{off}\times OEm$$ (6)

$$\text{Inhibition }\left(\%\right)=\frac{OEm}{{Em}_{free}+OEm}\times 100$$ (7)

**TABLE 2:** Comparisons of the geometric mean values between predicted and observed PK variables in different population ancestry.

Ancestry	Dosing regimen	Population	Age range (year)	Proportion of female	Parameters	PBPK prediction	Observed value	Fold-error	Clinical study
Caucasian	20 mg, SD	Healthy	21–53	50%	C_max_ (nmol/L)	32.7 (26.2–39.3)	31.6 (18.9–55.2)	1.03	Planchard et al. (2016)
					AUC (nmol·h/L)	1408 (970–1922)	1060 (607–1520)	1.33	
	80 mg, SD	Healthy	18–55	0	C_max_ (nmol/L)	134.4 (113.9–165.1)	126.1 (49.0–200)	1.07	Vishwanathan et al. (2018a)
					AUC (nmol·h/L)	7729 (3938–10930)	6269 (2670–14200)	1.23	
		Patients	31–84	68	C_max_ (nmol/L)	281.6 (212.4–384.9)	218.0 (95.2–381)	1.29	
					AUC (nmol·h/L)	20317 (11397–33406)	12530 (6050–25500)	1.62	
	80 mg, SD	Healthy	21–61	0	C_max_ (nmol/L)	131.2 (18.4%)	118.0 (28.1%)	1.11	Vishwanathan et al. (2019)
					AUC (nmol·h/L)	7487.2 (33.8%)	6791 (27.6%)	1.10	
	80 mg, OD for 29 days	Patients	44–83	71	C_ss,max_ (nmol/L)	577.3 (64.0%)	620.1 (34%)	0.93	Harvey et al. (2018)
					AUC_ss_ (nmol·h/L)	13643 (61.0%)	11530 (37%)	1.18	
	80 mg, SD	Patients	56–73	60	C_max_ (nmol/L)	254.1 (14.6%)	291.8 (45%)	0.87	Grande et al. (2019)
					AUC (nmol·h/L)	13698.3 (56.0%)	15780 (38%)	0.87	
Japanese	80 mg, SD	Patients	62.5 (median)	75	C_max_ (nmol/L)	295.3 (224.8–370.9)	198.3 (85.3–598.0)	1.49	Planchard et al. (2016)
					AUC (nmol·h/L)	11446 (8756.4–16499)	10590 (4940–25000)	1.08	
	160 mg, SD				C_max_ (nmol/L)	588.8 (448.2–740.8)	430.1 (236.0–813.0)	1.37	
					AUC (nmol·h/L)	22198 (16956–32089)	24610 (15600–39100)	0.90	
	240 mg, SD				C_max_ (nmol/L)	815.1 (648.5–1002.6)	458.7 (164.0–1040)	1.78	
					AUC (nmol·h/L)	32300 (24617–46849)	29360 (9920–59000)	1.10	
	20 mg, OD for 22 days				C_ss,max_ (nmol/L)	128.9 (103.5–172.8)	106.4 (45.4–280.0)	1.21	
					AUC_ss_ (nmol·h/L)	2402 (1853–3448)	1964 (871–4990)	1.22	
	40 mg, OD for 22 days				C_ss,max_ (nmol/L)	283.4 (225.6–38.1)	306.2 (127–807)	0.93	
					AUC_ss_ (nmol·h/L)	5802 (4432–8337)	5640 (2040–14100)	1.03	
	80 mg, OD for 22 days				C_ss,max_ (nmol/L)	542.4 (426.5–729.6)	623.8 (167–2100)	0.87	
					AUC_ss_ (nmol·h/L)	11727 (8921–16913)	11930 (3650–38900)	0.98	
	160 mg, OD for 22 days				C_ss,max_ (nmol/L)	988.8 (769.4–1316.8)	1255 (282–4760)	0.79	
					AUC_ss_ (nmol·h/L)	22111 (16801–32035)	23910 (5950–97000)	0.92	
	240 mg, OD for 22 days				C_ss,max_ (nmol/L)	1414.0 (1096.4–1901.2)	1491 (723–2620)	0.95	
					AUC_ss_ (nmol·h/L)	32332 (24535–46970)	28310 (1150–51200)	1.14	
	80 mg, OD, on day 15		53–78	69	C_ss,max_ (nmol/L)	620.7 (449.5–1017.0)	612 (510–882)	1.01	Fujiwara et al. (2023)
					AUC_ss_ (nmol·h/L)	11813.6 (6915–1998)	11000 (5100–15820)	1.07	
Chinese	40 mg, OD, on day 1	Patients	33–73	47	C_max_ (nmol/L)	164.3 (19.9%)	103.8 (79.2%)	1.58	Zhao et al. (2018)
					AUC (nmol·h/L)	6878 (62.9%)	6323 (55.8%)	1.09	
	40 mg, OD, on day 8				C_ss,max_ (nmol/L)	379.1 (37.0%)	303.4 (48.0%)	1.25	
					AUC_ss_ (nmol·h/L)	7243 (43.9%)	5698 (52.6%)	1.27	
	80 mg, OD, on day 1		35–76	69	C_max_ (nmol/L)	285.7 (13.9%)	195.9 (49.8%)	1.46	
					AUC (nmol·h/L)	13364 (70.1%)	10260 (33.1%)	1.30	
	80 mg, OD, on day 8				C_ss,max_ (nmol/L)	595.5 (51.4%)	550.4 (32.4%)	1.08	
					AUC_ss_ (nmol·h/L)	10637 (64.9%)	9570 (35.9%)	1.11	

SD, Single-dose; OD, once daily; AUC, Area under PK, curve; AUC_ss_, Area under PK, curve at steady state; C_max_, Peak concentration; C_ss_,_max_, Peak concentration at steady state; CL/F, apparent clearance; CL_ss_/F, apparent clearance at steady state.

Where OEm is the concentration of OSI-EGFRm^+^ complex formed. Em_free_ is the free concentration of EGFRm^+^. Em_0_ is starting concentration of EGFRm^+^, and was set 0.299 μM based on the paper (Bartelink et al., 2022). C_lung_, k_on_, k_off_, and k_turnover_ are the free OSI concentration in the lung, association, dissociation rate constant of OSI, and re-synthesis rate constant of EGFRm^+^, respectively. The k_on_ values of 0.91 (binding to T790M) and 0.44 (binding to L858R) μM^−1^s^−1^ were obtained from the paper (Zhai et al., 2020), equivalent to the ratio of K_inact_/K_i_. k_off_ was assumed to be 0 due to irreversible covalent binding to EGFR for OSI. k_turnover_ was obtained to be 0.025 h^−1^ from the paper (Greig et al., 2015).

### 2.4 Effect of the key factors on Plasma C_trough_ and EGFRm^+^ inhibition

The diseased PBPK model was used to evaluate the influence of CPY3A4 variants, CYP1A2 CL_int,u_, auto-inhibition/induction on CYP3A4, f_up_, and albumin level on plasma C_trough_ and pulmonary EGFRm^+^ inhibition. 26 CPY3A4 variant CL_int,u_ values from a referenced paper (Gao et al., 2022) were incorporated into the PBPK model to assess the impact. The CYP1A2 CL_int,u_ was varied within the range of 0.52–5.2 μL/min/pmol. Additionally, Auto-inhibition and induction were set at 0 for K_i_/E_max_ CYP3A4 and 2.5 μM (K_i_ CYP3A4)/10.8 (E_max_ CYP3A4), respectively. The f_up_ values were set within the range of 0.009–0.034, while the albumin levels were set within the range of 1.1–6.1 g/dL. The dosing regimen involved taking 80 mg MD for 14 consecutive days, with the assumption that steady-state is reached after 14 days of OSI intake. The virtual population’s demographic characteristics were set to Caucasian, as mentioned in Table 2.

### 2.5 Statistical analysis

In the analysis conducted using JMP software (JMP Pro 16.0.0, SAS Institute Inc., NC, United States), a one-way analysis of variance (ANOVA) was performed. The Tukey-Kramer test was used to compare multiple groups. A *p*-value of less than 0.05 and 0.01 was considered statistically significant, indicating a significant difference between the groups being compared.

## 3 Results

### 3.1 Development and validation of the PBPK model

According to Table 2, the ratios of predicted and observed geometric mean AUC and C_max_ for OSI were within the acceptable range of 0.5–2.0, indicating that the population PBPK model accurately predicts the PK variables of OSI at single-dose and steady-state in the three ethnic populations (Caucasian, Japanese, and Chinese). Moreover, it is notable that most of the predicted/observed ratios fell within the range of 0.8–1.25, further suggesting a high level of accuracy in the PBPK model’s predictions. This indicates that the model is capable of accurately capturing the PK behavior of OSI in individuals from different ethnic backgrounds, both in terms of OSI exposure (AUC, C_max_). Overall, the simulations validate the predictive performance of the PBPK model for OSI in healthy and diseased populations, also providing confidence in its ability to estimate PK parameters across diverse ethnic populations.

Additionally, the predicted ratios of PK variables for OSI co-administered with ITR and RIF are provided in Supplementary Table S1. These predicted ratios were found to be consistent with the clinical data (Vishwanathan et al., 2018b), further confirming the accuracy of the CYP3A4 metabolic contribution in the PBPK model.

### 3.2 Sensitivity analysis

According to Table 3, the most sensitive parameters with a significant impact on the AUC_ss_, C_ss_,_max_ and plasma C_trough_ of OSI are f_up_ and albumin level. The albumin level has a significant impact on all three PK variables, in particular on plasma C_trough_, with a SC value of −2.10. Similarly, f_up_ also has a significant impact on two PK variables. Additionally, CYP3A4 CL_int,u_, CYP3A4 concentration, and liver volume also exhibit a relatively large influence on PK variables. However, the majority of the modeling parameters had only a slight impact on the PK variables of OSI, as revealed by the sensitivity analysis.

**TABLE 3:** Sensitivity analysis of modelling parameters.

Modelling parameters	SC values		
	AUC_ss_	C_ss,max_	C_trough_
f_up_	−1.73	−0.91	−1.32
Rbp	0.05	0.05	0.08
GET	−0.02	−0.1	0.01
Albumin	−1.63	−1.34	−2.10
CYP1A2 CL_int,u_	−0.30	−0.16	−0.23
CYP2A6 CL_int,u_	−0.06	−0.04	−0.07
CYP2C9 CL_int,u_	−0.45	−0.25	−0.35
CYP2E1 CL_int,u_	−0.02	−0.01	−0.02
CYP3A4 CL_int,u_	−0.80	−0.47	−0.64
CYP1A2 concentration	−0.30	−0.16	−0.23
CYP2A6 concentration	−0.06	−0.04	−0.07
CYP2C9 concentration	−0.45	−0.25	−0.35
CYP2E1 concentration	−0.02	−0.01	−0.02
CYP3A4 concentration	−0.80	−0.47	−0.64
Liver volume	−0.64	−0.39	−0.51
CYP3A4 auto inhibition/induction	0.19	0.12	0.16

### 3.3 Plasma C_trough_ and EGFRm^+^ inhibition prediction

Table 4 displays the predicted plasma C_trough_ values for various dosing regimens in NSCLC patients of three ethnic groups, as determined by the PBPK model. The observed ratios ranged from 0.80 to 1.25, indicating a good agreement between the predicted and actual C_trough_ values. These simulations demonstrate that the diseased PBPK model is capable of accurately predicting steady-state OSI plasma C_trough_ in NSCLC patients.

**TABLE 4:** Comparisons of the geometric mean plasma C_trough_ between predicted and observed data in different population ancestry.

Clinical study	Ancestry	Dosing regimen	Predicted plasma C_trough_ (nmol/L)	Observed plasma C_trough_ (nmol/L)	Predicted/Observed
Harvey et al. (2018)	Caucasian	80 mg, MD	342.4 (53.3%)	381.7 (39.0%)	0.90
Planchard et al. (2016)	Japanese	20 mg, MD	62.9 (51.2%)	51.2 (36.0%)	1.23
		40 mg, MD	161.9 (49.1%)	179.3 (50.0%)	0.90
		80 mg, MD	350.5 (54.5%)	386.4 (56.6%)	0.91
		160 mg, MD	699.2 (55.8%)	784.4 (62.8%)	0.89
		240 mg, MD	973.4 (64.1%)	929.1 (55.4%)	1.05
Fujiwara et al. (2023)	Japanese	80 mg, MD	372.2 (64.2%)	346.3 (32.9%)	1.07
Zhao et al. (2018)	Chinese	40 mg, MD	198.0 (48.7%)	183.0 (60%)	1.08
		80 mg, MD	366.3 (46.1%)	318 (43%)	1.15

**FIGURE 1:**
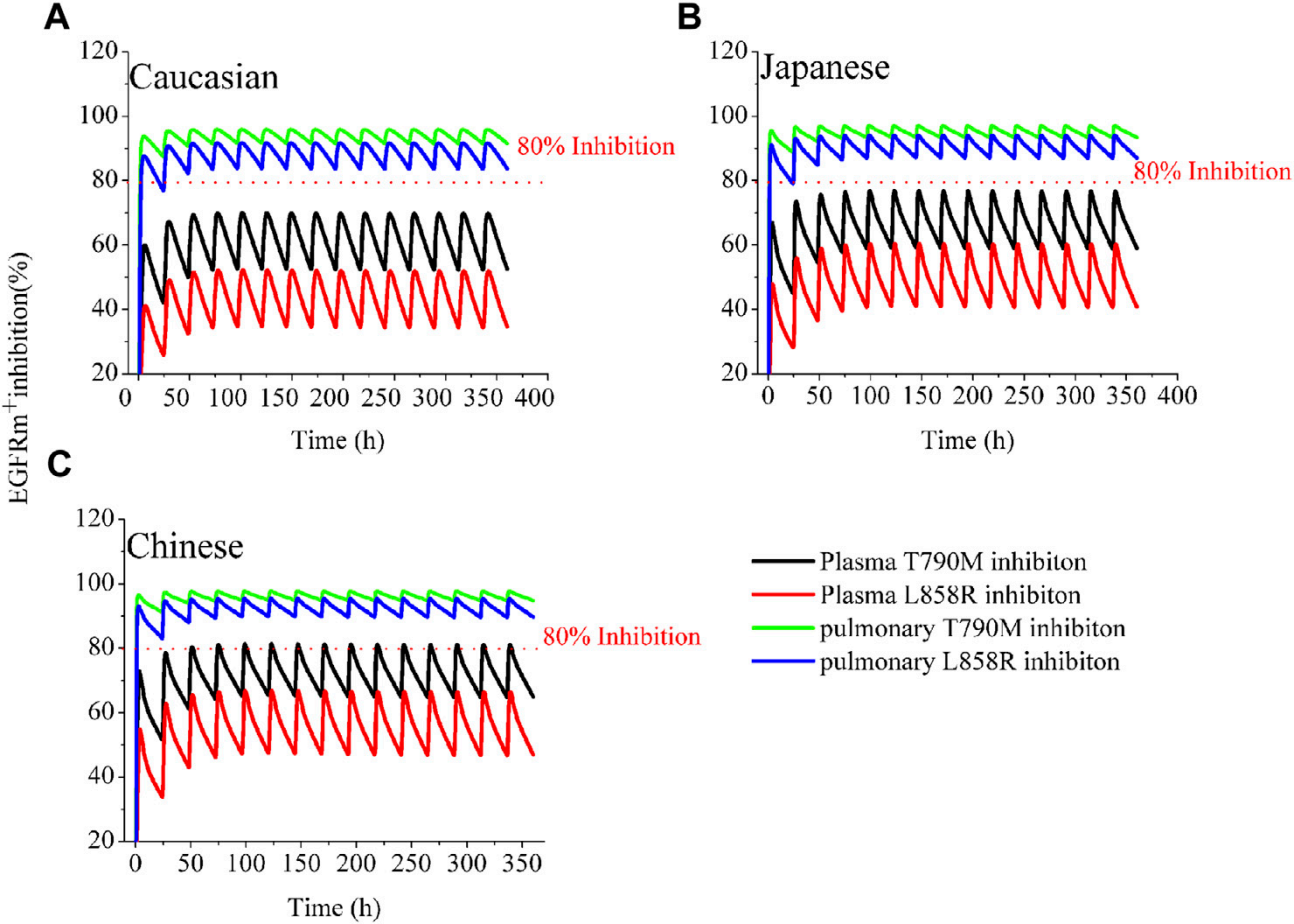
Simulations of EGFRm+ inhibition in plasma and lung in the three ethnic groups by OSI. The time-course of plasma and pulmonary EGFRm^+^ inhibition in Caucasian **(A)**, Japanese **(B)**, and Chinese **(C)** following 80 mg OD for 14 consecutive days. The blue and green solid lines represent pulmonary EGFRm^+^ inhibition; the black and red solid lines represent plasma EGFRm^+^ inhibition.

Figure 1 illustrates the time profiles of plasma and pulmonary EGFRm^+^ (T790M and L858R mutants) inhibition in the three ethnic groups during a 14-day period of treatment with 80 mg MD of OSI. According to a study on the effect of EGFRm^+^ suppression (Food and Drug Administration FDA, 2015), an effective pharmacodynamics (PD) threshold for OSI was defined as more than 80% EGFRm^+^ inhibition. The time-course of plasma and pulmonary T790M and L858R inhibition is similar across the three ethnic groups. Notably, the minimal rates of pulmonary inhibition for both mutants exceed 80%, which is significantly higher than in plasma. The simulations of pulmonary 80% inhibition align well with a study conducted on NCI H1975 cells (Food and Drug Administration FDA, 2015). As a result, the prediction of pulmonary T790M and L858R inhibition, as target tissue, over time proves valuable for assessing clinical efficacy compared to plasma EGFRm^+^ inhibition.

### 3.4 Assessment of inter-ethnic differences and Plasma C_trough_ variability

#### 3.4.1 Apparent CL/F simulations of OSI in healthy and diseased populations

**FIGURE 2:**
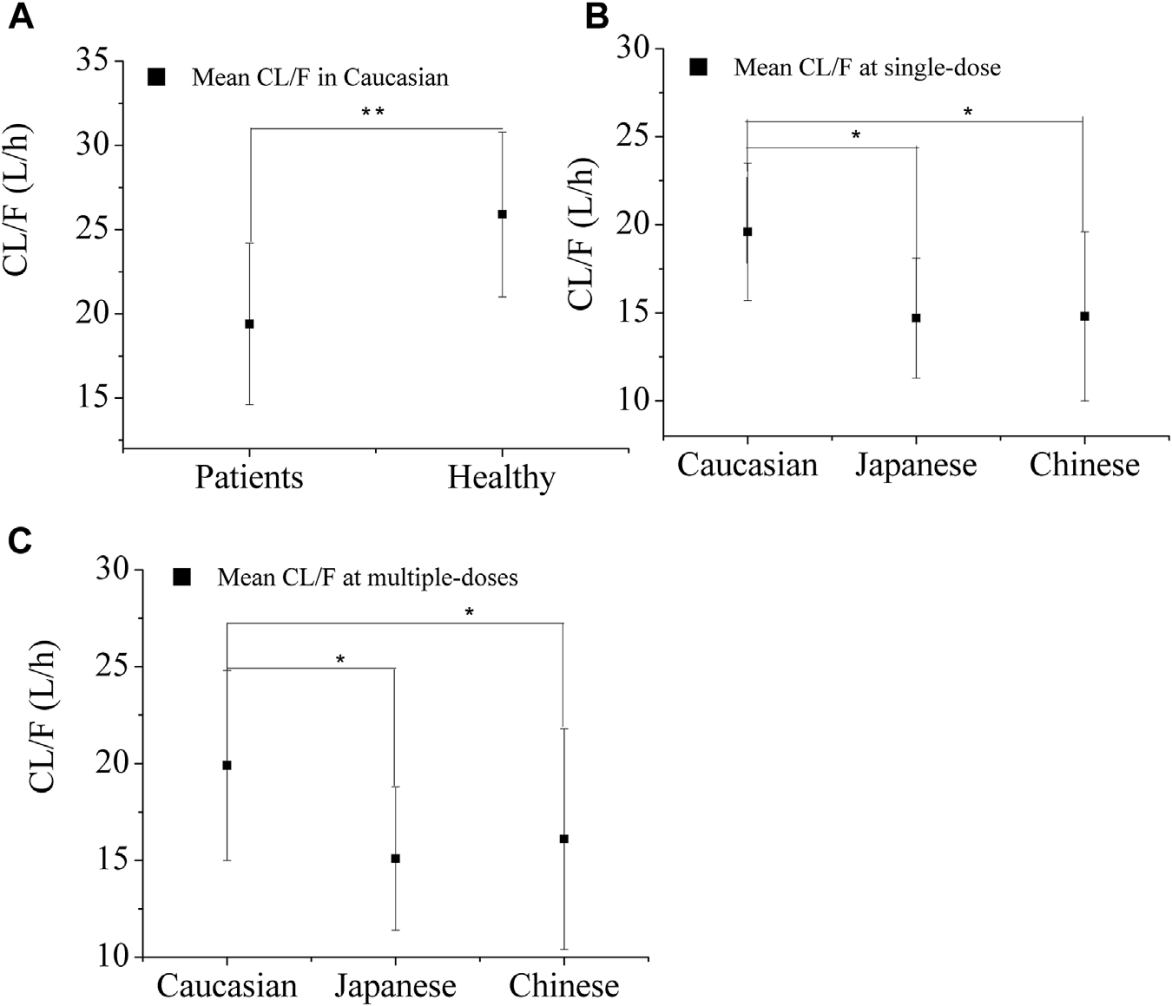
Predicted apparent clearance (CL/F) of OSI in the three ethnic groups for healthy Caucasian **(A)** and Patients **(B and C)** after single-dose **(B)** and multiple-doses **(C)**. Note:* and ** denotes statistically significant difference, *p* < 0.05 and *p* < 0.01.

Figure 2A illustrates the predicted CL/F of OSI in both healthy and diseased Caucasian populations. The CL/F in the healthy population is statistically significant higher (*p* < 0.01) compared to that in patients. This disparity can account for the observed clinical OSI exposure, which is approximately two times higher in Caucasian patients when compared to healthy individuals (Harvey et al., 2018; Vishwanathan et al., 2019). The difference in exposure between healthy and diseased populations is primarily attributed to variations in f_up_ and albumin levels. When the f_up_ and albumin levels in the diseased PBPK model were adjusted to the same values with the healthy PBPK model, an approximately 1.29-fold difference in exposure was observed between the two groups. This difference of 1.29-fold can be further explained by variations in CYP enzyme concentration between healthy and diseased populations. Moreover, the PBPK model prediction of relative contribution of the three factors to total CL/F of OSI is present in Supplementary Table S2.

Figures 2B, C displays the predicted apparent CL/F of OSI in the three ethnic groups. ANOVA analysis of the simulated CL/F values indicated a significant difference between Caucasian and Japanese patient s (*p*<0.05), as well as between Caucasian and Chinese patients (*p* < 0.05), for both single-dose (80 mg) and multiple-dose (80 mg) regimens. However, no significant difference was observed between Japanese and Chinese patients. The geometric mean CL/F in Caucasian patients was approximately 1.3-fold higher than in Japanese and Chinese patients for the single-dose regimen, and 1.2-fold lower for the multiple-dose regimen. The lower CL/F in Asian patients resulted in a lower AZ5104 AUC compared to Caucasian patients, indicating a 10%–23% decrease in AZ5104 AUC in Asian NSCLC patients relative to Caucasian patients (Brown et al., 2017).

#### 3.4.2 Effect of CPY3A4 variants on Plasma C_trough_ and EGFRm^+^ inhibition

**FIGURE 3:**
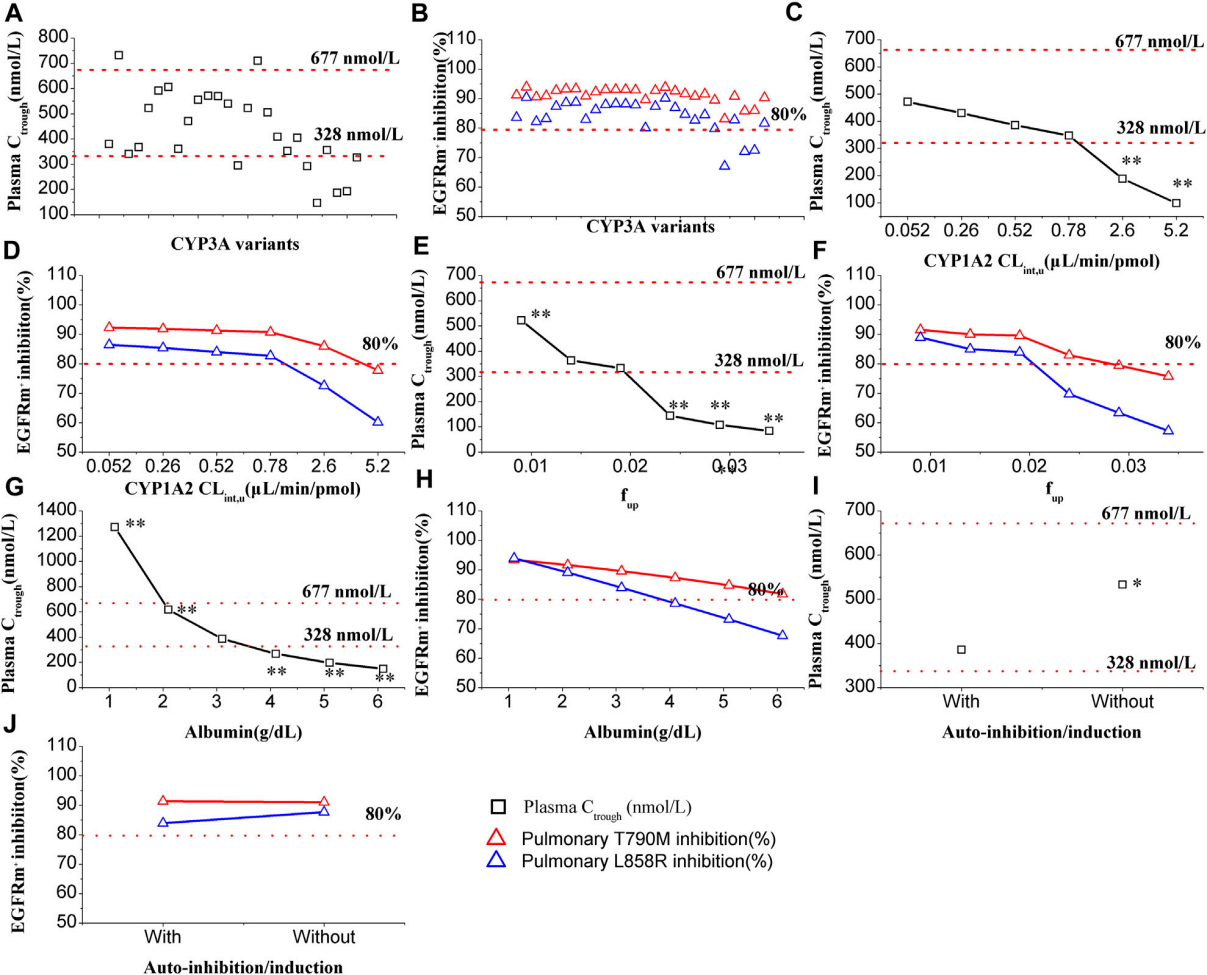
Effect of multiple factors on plasma C_trough_ and pulmonary EGFRm^+^ inhibition. Plasma C_trough_ values are affected by CYP3A4 variants **(A)**, CYP1A2 C_lint,u_ **(C)**, f_up_ **(E)**, albumin level **(G)**, and with or without auto-inhibition/induction **(I)**. EGFRm^+^ inhibition values are affected by CYP3A4 variants **(B)**, CYP1A2 C_lint,u_ **(D)**, f_up_ **(F)**, albumin level **(H)**, and with or without auto-inhibition/induction **(J)**. One-way ANOVA was performed with the Tukey test. Note:*, ** denotes statistically significant difference, *p* < 0.05 and *p* < 0.01, respectively.

In Figure 3A, it is evident that CYP3A4 variants have a significant impact on the plasma C_trough_ of OSI. The most substantial decrease in OSI plasma C_trough_ was observed with the CYP3A4.29 variant, which resulted in approximately 61% lower plasma C_trough_ levels compared to the wild type. Conversely, the maximal increase in plasma C_trough_ was seen with CYP3A4.2 variants, where the plasma C_trough_ were approximately 1.93-times higher than the wild type. Among these CYP3A4 variants, two plasma C_trough_ values (CYP3A4.2/17) exceeded the PK threshold for safety, while six C_trough_ values (CYP3A4.15/28/29/32/33/34) fell below the effective PK threshold. On the other hand, only slight changes were observed in pulmonary EGFRm^+^ E790M inhibition, within a range of 15% compared to the wild type. However, three inhibition values against L858R were below the 80% inhibition threshold (Figure 3B).

#### 3.4.3 Effect of CPY1A2 activity on C_trough_ and EGFRm^+^ inhibition

In Figure 3C, the influence of CYP1A2 activity on the plasma C_trough_ of OSI is demonstrated. The simulations indicate that CYP1A2 activity has a statistically significant impact (*p* < 0.01) on the plasma C_trough_ of OSI at tow data points. Specifically, when the CYP1A2 CL_int,u_ increased to 5- and 10-fold higher than the original value (0.52 μL/min/pmol) in the model, the two plasma C_trough_ values were below 328 nmol/L of PK effective threshold. On the other hand, in Figure 3D, it can be observed that pulmonary T790M inhibition is less affected by CYP1A2 activity. However, for pulmonary L858R inhibition, two data points were below the 80% inhibition threshold when the CYP1A2 CL_int,u_ increased to 5- and 10-fold higher compared to the original value.

#### 3.4.4 Effect of f_up_ on C_trough_ and EGFRm^+^ inhibition

In Figure 3E, the influence of f_up_ on the plasma C_trough_ of OSI is shown. The simulations demonstrate that f_up_ has a highly statistically significant impact on the plasma C_trough_ of OSI, with four values having *p* < 0.01. As f_up_ increases, the plasma C_trough_ gradually decreases, and three data points fall below the PK efficacy threshold. Similarly, in Figure 3F, a significant influence of f_up_ is observed in both pulmonary T790M and L858R inhibition. Specifically, for pulmonary L858R inhibition, three data points were below the 80% inhibition PD threshold.

#### 3.4.5 Effect of albumin level on C_trough_ and EGFRm^+^ inhibition

In Figure 3G, the influence of albumin levels on the plasma C_trough_ of OSI is depicted. The simulations reveal that albumin levels have an extremely significant impact on the plasma C_trough_ of OSI. The ANOVA analysis of the simulated data indicates that all values differ statistically significantly compared to the original level (0.31 g/dL) in the model. Additionally, four data points fall outside the range of efficacy and safety PK thresholds, suggesting inadequate clinical efficacy and potential safety. Similarly, in Figure 3H, a significant impact of albumin levels is observed on pulmonary EGFRm^+^ inhibition. As albumin levels increase, there is a gradual decrease in EGFRm^+^ inhibition, with several data points falling below 80% for L858R inhibition.

#### 3.4.6 Effect of auto-inhibition/induction C_trough_ and EGFRm^+^ inhibition

In Figure 3I, the influence of auto-inhibition/induction on the plasma C_trough_ of OSI is presented. The simulations indicate that auto-inhibition/induction towards CYP3A4 has a statistically significant impact (*p* < 0.05) on the plasma C_trough_ of OSI. However, this impact remains within the range of efficacy and safety PK thresholds, suggesting that it may not significantly compromise the effectiveness or safety of the OSI. On the other hand, in Figure 3J, only a slight impact on pulmonary EGFRm^+^ inhibition is observed due to auto-inhibition/induction. This suggests that changes in CYP3A4 activity resulting from auto-inhibition/induction have minimal effects on the inhibition of pulmonary EGFRm^+^ inhibition.

#### 3.4.7 Key factors of affecting the PK variability of inter-patients

**FIGURE 4:**
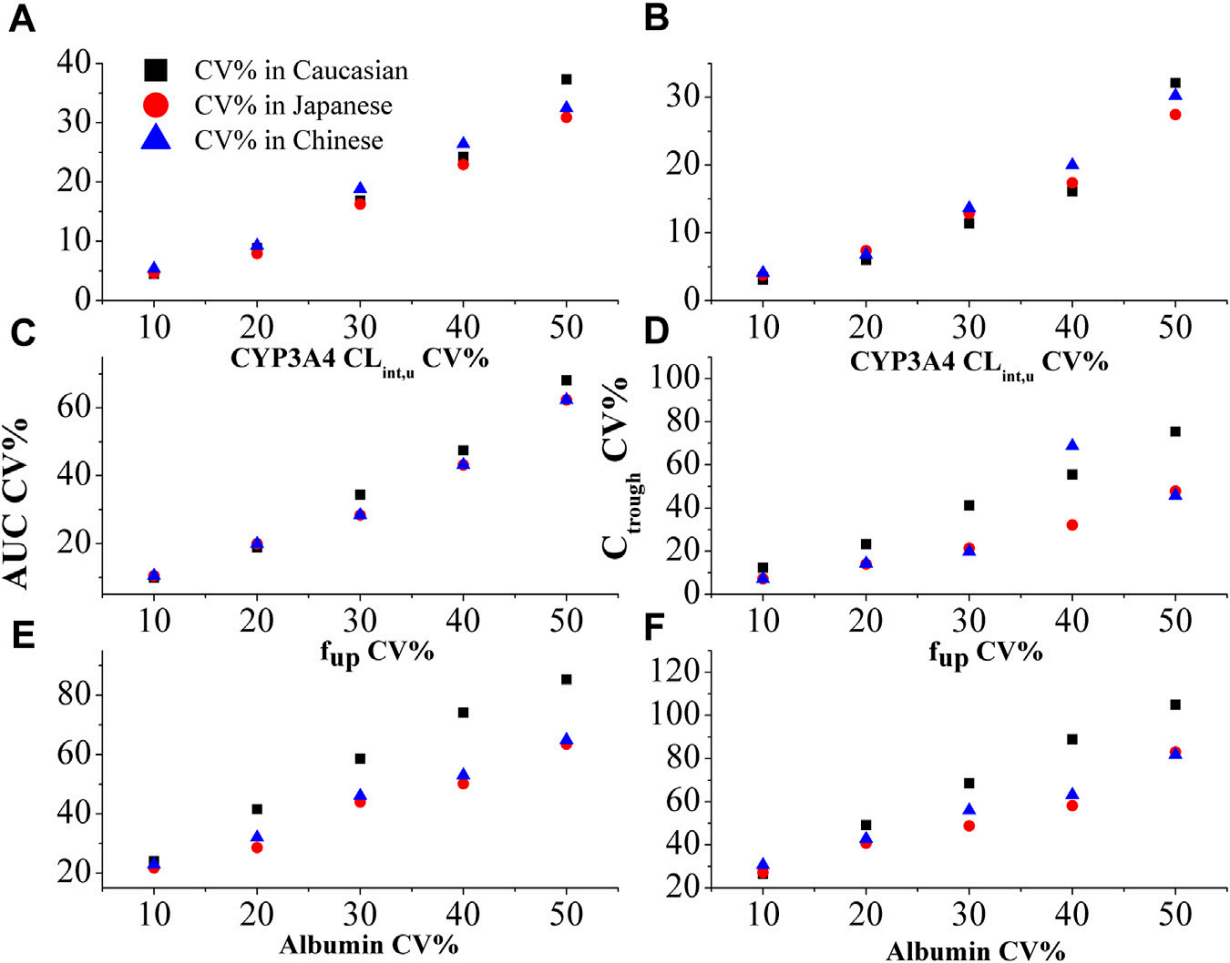
The effect of %CV of CYP3A4 CL_int,u_ **(A and B)**, f_up_ **(C and D)**, and albumin level **(E and F)** on the variability in AUC and plasma C_trough_ in three ethnic populations.

Figure 4 demonstrates the impact of %CV of CYP3A4 CL_int,u_, %CV of f_up_, and %CV of albumin level on the PK variability of inter-patients. The PBPK model simulations reveal that the %CV of albumin level primarily contributes to the PK variability among patients (as shown in Figures 4E, F). Notably, the influence of %CV of albumin on PK variability is more prominent in Caucasian than in Japanese, and Chinese populations. Specifically, it is observed that a 20% CV of albumin in Caucasian and 30% CV in Japanese and Chinese populations can result in an approximate 50% CV of PK variability among patients. Considering the diseased PBPK model, it is reasonable to set the %CV of albumin level at 20% in Caucasians and 30% in Japanese and Chinese populations to account for the observed PK variability.

### 3.5 Optimal dosage recommendation based on the PBPK model

**FIGURE 5:**
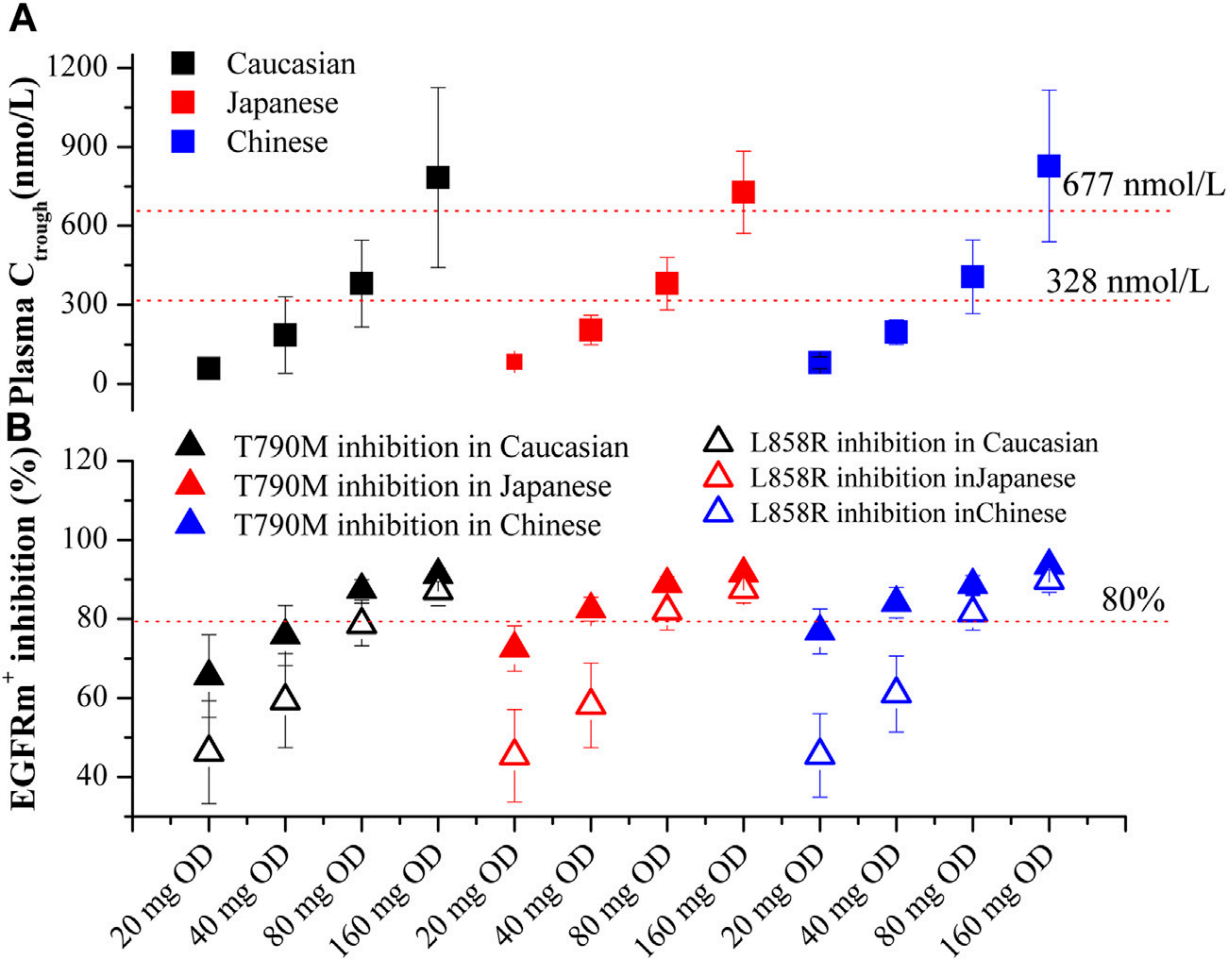
PBPK simulations of OSI plasma C_trough_ **(A)** and pulmonary EGFRm^+^ inhibition **(B)** in three ethnic populations. Data were shown as geometric mean values ±95% CI. In Figure 4B, Solid Square and Open Square denote minimal inhibition against T790M and L858R mutants, respectively.

Based on Figures 5A, B, the plasma C_trough_ and pulmonary EGFRm^+^ inhibition in three ethnic populations were evaluated for four dosing regimens of OSI. The optimal dosing regimens for each ethnic group were determined based on the geometric mean of C_trough_ and minimal EGFRm^+^ inhibition (Calculated using pulmonary C_trough_), along with a 95% confidence interval (95% CI). The results show that the plasma C_trough_ values at the dose of 80 mg OD in all three ethnic NSCLC patients fall within the desired PK threshold range. This ensures both clinical efficacy (C_trough_ > 338 nmol/L) and safety (Ct_rough_ < 677 nmol/L). Additionally, the minimal EGFRm^+^ inhibition achieved at the doses of 80 mg and 160 mg OD in all three ethnic NSCLC patients exceeds the desired 80% inhibition. Moreover, it is observed that the minimal EGFRm^+^ inhibition approaches 100% at the dose of 80 mg OD compared to 160 mg OD. This suggests that higher dosing regimens may have limited increases in clinical efficacy, and result in more adverse event. Therefore, the recommended dose of 80 mg OD in NSCLC patients, as suggested by the PBPK model, aligns well with the clinical dose suggestion.

## 4 Discussion

This study successfully developed the PBPK models for OSI in healthy and diseased populations across three ethnic groups. These models were utilized to simulate the plasma C_trough_ and time-profiles of pulmonary EGFRm^+^ inhibition (specifically T790M and L858R mutants). The accuracy of the PBPK predictions was supported by multiple clinical PK studies (provided in Table 2/4) conducted on different ethnic populations and a clinical DDI study (Supplementary Table S1). Furthermore, the study investigated the impact of five factors on OSI plasma C_trough_ and pulmonary EGFRm^+^ inhibition. Additionally, the research aimed to identify key factors influencing the inter-patient and inter-ethnic PK variability of OSI. Notably, this study is the first to assess the PK and EGFRm^+^ inhibition of OSI in three ethnic populations while also evaluating the crucial factors that contribute to the PK variability of OSI.

### 4.1 Simulation of OSI distribution and EGFRm^+^ inhibition

The K_lu,p_ parameter plays a crucial role in characterizing the lung distribution of OSI from the plasma. According to the Rodgers and Rowland approach, the predicted K_lu,*p*_-value was 14.3. However, experimental analysis revealed a mean K_lu,*p*_-value of 28.5 (Dickinson et al., 2016). The impact of the K_lu,*p*_-value on OSI plasma and lung PK is detailed in Supplementary Table S3. The analysis revealed that the K_lu,*p*_-value has a significant impact on lung PK but hardly impact on plasma PK. Consequently, in this model, a K_lu,*p*_-value of 28.5 was ultimately utilized to enhance the accuracy of lung concentration prediction.

### 4.2 Key factors of affecting the plasma C_trough_ and pulmonary EGFRm^+^ of OSI in PBPK model

In this study, the PBPK model was employed to simulate the impact of key parameters on OSI plasma C_trough_ and pulmonary EGFRm^+^ inhibition. The simulations revealed that CYP3A4 variants, f_up_, and albumin levels significantly influence these PK and PD outcomes. Sensitivity analysis further emphasized that f_up_ and albumin level are the most sensitive parameters affecting the PK variables of OSI. Given the significance of three parameters, adjustments were made in the diseased PBPK model. As no differences in f_up_ were observed among the different ethnic groups, the same f_up_ value was utilized for the PBPK model across all three ethnic populations. However, hypoalbuminemia is commonly observed in NSCLC patients and those with hepatic impairment, leading to a decrease in albumin levels. This decrease can substantially alter the plasma protein binding of OSI, which has a high affinity for albumin. The simulations conducted in this study strongly demonstrated that albumin level has a substantial impact on the PK variables of OSI as well as EGFRm^+^ inhibition. These findings align well with multiple clinical observations where albumin levels in patients have shown a strong correlation with clinical efficacy (Brown et al., 2017; Yokota et al., 2022; Ishikawa et al., 2023).

In this study, despite CYP3A4 CL_int,u_ not showing high sensitivity in the sensitivity analysis, it still exhibits a large impact on OSI plasma C_trough_ as shown in Figure 3A. Additionally, the effect of CYP1A2 activity on OSI (a CYP1A2 substrate) plasma C_trough_ and pulmonary EGFRm^+^ inhibition was also examined, considering that smoking can induce CYP1A2 activity. The simulation demonstrated that significant changes occur in OSI plasma C_trough_ and pulmonary EGFRm^+^ inhibition when CYP1A2 activity is increased by 5- or 10-fold. On the other hand, OSI plasma C_trough_ and pulmonary EGFRm^+^ inhibition exhibited a decreasing trend but no significant change when CYP1A2 activity increased to 1.5-fold (approximately 1.55-fold higher in enzyme activity caused by smoking). This finding aligns with multiple clinical observations where there is no significant impact on the clinical efficacy between smokers and nonsmokers (Food and Drug Administration FDA, 2015; Kishikawa et al., 2020; Jin et al., 2022; Yokota et al., 2022), despite two clinical studies suggesting a potential impact (Rodier et al., 2022; Abu Hamdh and Nazzal, 2023).

It's worth noting that OSI inhibits its own metabolism through competitive inhibition against CYP3A4 and also enhances its own metabolism through induction of CYP3A4 expression. Although the sensitivity analysis did not show significant effects of auto-inhibition and induction parameters on PK variables of OSI (as seen in Table 3), statistically significant changes in plasma C_trough_ were observed with or without auto-inhibition and induction (Figure 3I). Hence, it is necessary that auto-inhibition/induction parameters were incorporated into the PBPK model of OSI.

Overall, this study highlights the crucial role of several factors in influencing the plasma C_trough_ and pulmonary EGFRm^+^ inhibition of OSI, particularly in diseased populations. The study supports the clinical relevance of f_up_ and albumin levels in predicting OSI efficacy. Additionally, the CYP3A4 and CYP1A2 activities also play relative important roles in determining OSI plasma C_trough_ and pulmonary EGFRm^+^ inhibition. The impact of smoking-induced CYP1A2 activity on OSI appears to be less significant compared to the influence of CYP3A4 activity. Additionally, the competitive inhibition and induction of CYP3A4 by OSI may contribute to changes in plasma C_trough_, even though these effects were not prominently reflected in the sensitivity analysis.

In addition, the study revealed that T790M inhibition is less impacted by multiple factors compared to L858R inhibition. This disparity is primarily attributed to the larger k_on_ value of OSI against T790M than L858R (0.91 μM^−1^s^−1^ vs. 0.44 μM^−1^s^−1^). These key factors equally influence the plasma concentration of OSI, potentially resulting in smaller variations in pulmonary T790M inhibition due to the larger k_on_ value.

### 4.3 Efficacy and safety PK and PD thresholds defining

In certain clinical studies (Brown et al., 2017; Boosman et al., 2022; Hashino et al., 2023), when patients’ plasma C_trough_ levels for OSI were divided into only two groups, the relationship between exposure and efficacy was not observed. This is likely because clinical efficacy requires a minimal plasma C_trough_, while excessive C_trough_ levels can lead to adverse events and result in shorter progression-free survival (PFS). However, in a specific clinical study (Abu Hamdh and Nazzal, 2023), when patients’ plasma C_trough_ levels were divided into four groups, it was found that a C_trough_ level above 328 nmol/L was associated with longer PFS, whereas a C_trough_ level below 677 nmol/L could avoid some adverse events and also induce longer PFS. Therefore, for OSI, defining the range of 328 nmol/L to 677 nmol/L for efficacy and safety PK seems appropriate. However, the exact target inhibition level for clinical efficacy is not yet fully defined. In some relevant papers, thresholds such as >90% inhibition for soluble epoxide hydrolase (Lee et al., 2019), >70% inhibition for α-Glucosidase (Wang et al., 2019), and >75% inhibition for ALK (Yamazaki, 2013) have been established. Considering these references, in this study, a duration of >80% pulmonary EGFRm^+^ inhibition was selected as the efficacy PD threshold. This choice aligns with a study on NCI H1975 cells (Food and Drug Administration FDA, 2015).

### 4.4 PK variability between healthy and patient populations, inter-patient, and inter-ethnic group for OSI

In NSCLC patients, the exposure and CL/F of OSI are much lower compared to healthy subjects. The f_up_, albumin level, and metabolizing enzyme concentration may contribute to a significant increase in exposure among patients compared to healthy individuals (Figure 3). There is significant variability observed in OSI plasma concentration among individuals, with some patients showing more than 50% and even up to 80% variability (Planchard et al., 2016). Additionally, plasma exposure to AZ5104 is reported to be 10%–23% higher in Caucasian individuals compared to Asians (Brown et al., 2017). To account for these variabilities, the PBPK model analyzes key factors that influence the PK of OSI (as shown in Figures 2–4). The higher CL/F observed in Caucasians compared to Japanese and Chinese individuals is likely a main contribution of differences in healthy subjects and NSCLC patients (Figure 2). The observed higher CL/F in Caucasians may be linked to the greater abundance of CYP enzymes and larger liver volume in this population. Sensitivity analysis has shown that CYP3A4 concentration and liver volume have a relatively significant impact on AUC. (Table 3). The higher CL/F in healthy subjects may result from the difference in f_up_, CYP enzyme abundance and albumin level in the two populations. The inter-patient PK variability can be attributed to variations in %CV of albumin levels and metabolism enzyme variants among different patients (Figure 4). The %CV of albumin levels is approximately 20% CV in Caucasians and 30% CV in Japanese and Chinese populations, leading to a 50% PK variability among patients (Figure 4). Moreover, The PK variability in inter-ethnic group may be attributed to metabolism difference of CYP enzyme in the three inter-ethnic groups.

### 4.5 The PBPK model suggesting optimal dosage of OSI

In this study, the optimal dosage regimens of OSI for each ethnic population were determined based on the geometric mean and 95% CI of plasma C_trough_ and pulmonary EGFRm^+^ inhibition (Figure 5). These values were assessed to ensure they fell within the range of efficacy and safety PK/PD thresholds. This approach builds upon strategies proposed in previous studies (Johnson et al., 2019) and utilized in another research paper (Einolf et al., 2017). The PBPK model for patient population suggested that a dosage of 80 mg OD for OSI can achieve desirable values for both efficacy and safety based on plasma C_trough_. Additionally, pulmonary EGFRm^+^ inhibition was observed to be above 80% at both 80 mg and 160 mg OD dosages. Furthermore, the 95% CI values for T790M inhibition (a specific mutation) in the three ethnic groups were found to be above 80%, indicating a favorable therapeutic response.

### 4.6 The PBPK model limitations

The present model has several limitations that should be acknowledged. Firstly, the time-course of pulmonary EGFRm^+^ inhibition by OSI could not be directly verified using human clinical study data, which limits the ability to fully validate the model’s predictions in this regard. Secondly, the two active metabolites of OSI have not been incorporated into the current PBPK approach, which may lead to incomplete representation of the potential EGFRm^+^ inhibition. This could affect the accuracy of the model’s predictions. Additionally, it is worth noting that the mean value of albumin levels was used in the PBPK model due to the lack of experimentally determined albumin data in multiple PK studies. However, individual variations in albumin levels can exist, and not accounting for this variability may introduce some errors or inaccuracies in the predictions of plasma concentrations and PK parameters.

## 5 Conclusion

In conclusion, this study effectively developed and validated PBPK models for OSI in both healthy individuals and patient populations from three different ethnic groups. The models provided valuable insights into the PK and EGFRm^+^ inhibition profiles across these populations. Furthermore, the study explored the key factors that contribute to inter-patient variability and the differences in PK between healthy individuals and patients, as well as between different ethnic groups. By addressing these important aspects, this study advances our understanding of the PK characteristics of OSI and provides a foundation for optimizing dosage regimens for different patient populations. The insights gained from this research have the potential to guide personalized treatment approaches and improve the therapeutic outcomes of OSI in diverse patient populations.

## Data Availability

The original contributions presented in the study are included in the article/Supplementary Material, further inquiries can be directed to the corresponding authors.
