# Supplementary material for: Exploring inter-ethnic and inter-patient variability and optimal dosing of osimertinib: a physiologically based pharmacokinetic modeling approach
Source: Front Pharmacol. 2024 Mar 4;15:1363259. doi: 10.3389/fphar.2024.1363259 (PMC10946252; doi:10.3389/fphar.2024.1363259)
Supplement: Supplementary file 1 [file Table1.DOCX]

**Supplementary Table S1** The ratio of PK variables change of OSI in DDIs

| Variables | OSI only  (80 mg OD) | OSI + ITR  (80 mg OD+200 mg BID) | Predicted ratio | Observed ratio |
| --- | --- | --- | --- | --- |
| C_ss,max_ (nmol/L) | 213.8 | 225.6 | 1.06 | 0.83 |
| AUC_ss_ (nmol·h/L) | 9899.5 | 11483.5 | 1.11 | 1.26 |
| Parameters | OSI only  (80 mg OD) | OSI + RIF  (80 mg OD+600 mg OD) | Predicted ratio | Observed ratio |
| C_ss,max_ (nmol/L) | 538.6 | 175.2 | 0.33 | 0.26 |
| AUC_ss_ (nmol·h/L) | 10289 | 3355.8 | 0.33 | 0.20 |

**Supplementary Table S2** PBPK model prediction of relative contribution of f_up_, albumin levels and CYP 3A4 to total CL/F of OSI in different populations at a single-dose of 80 mg

| Factors | Total CL/F percentage (%) | | |
| --- | --- | --- | --- |
|  | Caucasian | Japanese | Chinese |
| f_up_ | 32.4 | 33.3 | 34.6 |
| Albumin | 48.6 | 51.9 | 46.2 |
| CYP concentration | 13.5 | 11.1 | 11.5 |

**Supplementary Table S3** The effect of K_lu,p_ on plasma and lung PK for OSI in Caucasian patients at 80 mg

| K_lu,p_ | PK parameters | | | |
| --- | --- | --- | --- | --- |
|  | Plasma | | Lung | |
|  | AUC(nmol·h/L) | C_max_ (nmol/L) | AUC (μmol·h/L) | C_max_ (nmol/L) |
| 14.3 | 13200 | 202.0 | 109 | 1970.4 |
| 28.5 | 13200 | 200.8 | 211 | 3796.8 |
