# Supplementary material for: Exploring inter-ethnic and inter-patient variability and optimal dosing of osimertinib: a physiologically based pharmacokinetic modeling approach
Source: Front Pharmacol. 2024 Mar 4;15:1363259. doi: 10.3389/fphar.2024.1363259 (PMC10946252; doi:10.3389/fphar.2024.1363259)
Supplement: Supplementary file 2 [file DataSheet1.docx]

**Single-dose**

Inhibition(%)=OEm/(Em_free_+OEm)*100

d/dt(OEm)= k_on_*C_lung_*f_u_*Em_free_-k_off_*OEm

INIT OEm=0

Ttotal= Em_free_ + OEm

d/dt(Em_free_)=Em_0_*k_turnover_- Em_free_*k_turnover_-k_on_*C_lung_*f_u_*Tfree+Koff*TC

INIT Tfree=0.3

T0 =0.3

Kturnover=0.025

kdeg=0.025

d/dt(Cp)= k12*Cc-k21*Cp

INIT Cp=0

d/dt(Aa)=-ka*Aa

INIT Aa=2.5

**Repeated-doses**

TO=TC/Ttotal*100

d/dt(Cc)=ka*Aa/Vc-kel*Cc-k12*Cc+k21*Cp-kon *fu*Cc*Tfree+Koff*TC

ka=0.51

kel=0.5

k12=0.5

k21=0.5

Vc=6

fu=0.019

kon=13500

koff=0.00

INIT Cc=0

d/dt(TC)= kon*Cc*fu*Tfree-Koff*TC

INIT TC=0

Ttotal= Tfree+TC

d/dt(Tfree)=T0*kturnover-Tfree*kdeg-kon*Cc*fu*Tfree+Koff*TC

INIT Tfree=0.299

T0 =0.299

Kturnover=0.025

kdeg=0.025

d/dt(Cp)= k12*Cc-k21*Cp

INIT Cp=0

d/dt(Aa)=pulse(0.2,0,24)-ka*Aa (肺=1)

INIT Aa=0
